# Alpha and beta EEG power reflects L-dopa acute administration in parkinsonian patients

**DOI:** 10.3389/fnagi.2014.00302

**Published:** 2014-11-05

**Authors:** Jean-Marc Melgari, Giuseppe Curcio, Francesca Mastrolilli, Gaetano Salomone, Laura Trotta, Mario Tombini, Lazzaro di Biase, Federica Scrascia, Rita Fini, Emma Fabrizio, Paolo Maria Rossini, Fabrizio Vernieri

**Affiliations:** ^1^Department of Neurology, Campus Bio-Medico UniversityRome, Italy; ^2^Department of Life, Health and Environmental Sciences, University of L’AquilaL’Aquila, Italy; ^3^Casa di Cura S. RaffaeleCassino, Italy; ^4^Department of Geriatrics, Neuroscience and Orthopedics, Catholic UniversityRome, Italy

**Keywords:** Parkinson’s disease, levodopa, quantitative EEG, power spectrum analysis, alpha rhythm, beta rhythm

## Abstract

**Aim**: To evaluate the effect of an acute L-dopa administration on eye-closed resting state electroencephalographic (EEG) activity of cognitively preserved Parkinsonian patients.

**Methods**: We examined 24 right-handed patients diagnosed as uncomplicated probable Parkinson’s disease (PD). Each patient underwent Unified Parkinson’s Disease Rating Scale (UPDRS)-part-III evaluation before and 60 min after an oral load of L-dopa-methyl-ester/carbidopa 250/25 mg. Resting condition eyes-closed EEG data were recorded both pre- and post L-dopa load. Absolute EEG power values were calculated at each scalp derivation for Delta, Theta, Alpha and Beta frequency bands. UPDRS scores (both global and subscale scores) and EEG data (power values of different frequency bands for each scalp derivation) were submitted to a statistical analysis to compare Pre and Post L-Dopa conditions. Finally, a correlation analysis was carried out between EEG spectral content and UPDRS scores.

**Results**: Considering EEG power spectral analysis, no statistically significant differences arose on Delta and Theta bands after L-dopa intake. Conversely, Alpha and Beta rhythms significantly increased on centro-parietal scalp derivations, as a function of L-dopa administration. Correlation analysis indicated a significant negative correlation between Beta power increase on centro-parietal areas and UPDRS subscores (Rigidity of arms and Bradykinesia). A minor significant negative correlation was also found between Alpha band increase and resting tremor.

**Conclusions**: Assuming that a significant change in EEG power spectrum after L-dopa intake may be related to dopaminergic mechanisms, our findings are consistent with the hypothesis that dopaminergic defective networks are implicated in cortical oscillatory abnormalities at rest in non-demented PD patients.

## Introduction

Parkinson’s disease (PD) is a movement disorder finding its origin from the degeneration of pigmented dopaminergic neurons within the substantia nigra pars compacta and their striatal projections (Rodriguez-Oroz et al., 2009). The core clinical features of PD—akinesia/bradykinesia, rigidity and tremor—are directly connected to this dopaminergic loss, as clearly documented by motor improvement consequent to dopamine replacement therapy (DRT; Yahr et al., 1969).

The progressive deterioration of the nigrostriatal dopaminergic system in PD leads to a secondary disruption in looping circuits constituted by cortico-basal ganglia-thalamo-cortical connections (Rodriguez-Oroz et al., 2009). Consequently, a functional alteration of brain oscillatory activity may be found in PD independently from cortical pathology and may be related to a dopaminergic loss.

Otherwise, according to Braak’s neuropathological staging system for PD (Braak et al., 2003), degeneration of non-dopaminergic nuclei (i.e., noradrenergic neurons in the locus coeruleus and serotoninergic neurons in the dorsal raphe nuclei) is a neuropathological hallmark that characterizes the earliest stages of PD. Since all these non-dopaminergic neurotransmitter systems are linked to corticopetal projections and regulate synaptic properties at cortical levels, they might also play a role in modifications of post-synaptic pyramidal cells membrane potentials, which represent the source of brain oscillatory electroencephalographic (EEG) activity.

Different studies investigated alterations in brain cortical oscillatory activity in PD by means of resting eye-closed EEG or magnetoencephalography (MEG). These techniques analyze brain activity by using electromagnetic properties of the brain and are characterized by a relatively low spatial resolution in the face of a high temporal resolution (in the order of milliseconds). Thus, compared to neuroimaging techniques (Functional Magnetic Resonance Imaging, fMRI; Positron Emission Tomography, PET), that explore hemodynamic properties of the brain with high spatial and low temporal resolution, they allow to assess changes in neuronal response in a brief time scale (Meyer-Lindenberg, 2010). Moreover, EEG, unlike MEG, is a portable and low-cost technique; in addition, the EEG detects the currents radial and tangential to the scalp, while MEG detects tangential current only. The EEG signal can be modulated by the effect of various neurotransmitters, but investigating the effect of single neurotransmitters on neural activity is not simple for an *in vivo*, non-invasive, study, because the various neurotransmitters are interconnected and in equilibrium with each other.

Most studies reported a widespread slowing of cortical rhythms with a higher amplitude of slower (theta and/or delta) frequencies in non-demented PD patients (Soikkeli et al., 1991; Neufeld et al., 1994; Gagnon et al., 2004; Bosboom et al., 2006). On the other hand, considered that alpha and beta frequencies constitute the leading characteristic of normal EEG activity at rest, a disruption of these rhythms might be interpreted as an EEG marker of altered cortical functioning and processing of information.

Nevertheless, all these studies failed to demonstrate which neurotransmitter system is predominantly involved in the generation of this altered brain oscillatory function and whether dopaminergic neurotransmission failure in PD may have a role in cortical dynamics. A few reports addressed this question, with controversial results. A previous quantitative EEG study demonstrated a topographically confined (left occipital cortex) increase in spectral power over all frequency bands, as a consequence of chronic L-dopa replacement therapy (Yaar and Shapiro, 1983). Authors concluded that their findings were consistent with the existence of dopaminergic mechanism in the generation of human EEG. On the other hand, a recent MEG study on non-demented PD patients (Stoffers et al., 2007) did not find any significant effect on spectral power with acute administration of L-dopa, postulating a role for a non-dopaminergic corticopetal neurotransmitter in spectral power changes.

An increase in the dose of L-dopa, in Parkinsonian patients, may induce periodic generalized triphasic waves on the EEG (Neufeld, 1992). Stanzione et al. (1996) demonstrated an increase in the delta power and a decrease in beta-1 power by comparing the EEG of Parkinsonian patients before and during L-dopa therapy. Amphetamines, cocaine and methylphenidate are central nervous system stimulants that potentiate dopamine activity. These drugs increase alpha and beta activities, and reduce delta/theta activities (Saletu, 1976; Nausieda, 1979; Herning et al., 1985).

In the present study, we investigated if DRT by means of L-dopa may acutely change resting-state oscillatory brain activity, assuming that a significant modification of EEG power spectrum might reflect a dopaminergic mechanism in its generation. In addition, we aimed to determine if EEG power spectrum modifications might correlate with changes in motor performances after an individual oral load of L-dopa.

## Materials and methods

### Participants

Twenty-four patients (Table 1), diagnosed as uncomplicated probable PD according to the UK PD Society Brain Bank diagnostic criteria (Gelb et al., 1999), were recruited in the study group from Neurology inpatient or outpatient clinic of Policlinico Campus Bio-Medico of Rome.

**Table 1:** **Demographic and clinical features of PD patients (mean ± standard deviation)**.

	PD patients (*n* = 24)
Age (y)	72.63 ± 6.85
Sex (M/F)	19/5
Education (y)	9.08 ± 3.66
Disease duration (y)	4.08 ± 3.62
Age at motor onset	68.54 ± 7.33
Tremorigen/rigid-akinetic	8/16
Side of initial symptoms (left/right)	17/7
Side of more predominant symptoms	17/7
Hoehn and Yahr stage	1.90 ± 0.64
UPDRS-III in off state	25.00 ± 9.51
UPDRS-III in on state	13.92 ± 6.82
MMSE	27.37 ± 2.80
LEDD	190.00 ± 311.75
Naïve/on dopamine replacement therapy	15/9

*Legend: n = number, y = years, M/F = male/female, UPDRS-III = Unified Parkinson’s Disease Rating Scale part III, MMSE = Mini Mental State Examination, LEDD = L-dopa equivalent daily dose (Grosset et al., 2004)*.

Exclusion criteria were the presence of other neurological conditions or the current use of psychoactive drugs that could alter EEG brain activity, the presence of ischemic cerebral white matter lesions on T2-weighted 1.5-Tesla MRI scans, and a history of head trauma or psychiatric disorders. Moreover, each patient underwent a complete neuropsychological evaluation (Rey Auditory Verbal Learning, recall of Rey-Osterrieth complex figure, backward and forward Digit span and Corsi test, oral denomination for objects of real life, and semantic and phonological verbal fluency, Raven’s Colored Progressive Matrices, digit visual search task, Rey-Osterrieth complex figure, Mini Mental State Examination-MMSE) to evaluate global cognitive efficiency (mean score at MMSE = 27.37 ± 2.80) and to exclude the coexistence of dementia.

Disease staging (mean score: 1.90 ± 0.64) was expressed by the Hoehn and Yahr (1967) grading scale.

The study protocol had the approval of the local Ethical Committee and all participants gave their written informed consent.

### Procedure

We collected clinical information about the progression of the disease and treatment [calculated as L-dopa equivalent daily dose (Grosset et al., 2004)]. Each patient was examined by a movement disorder expert and underwent a neurological examination, including the Unified Parkinson’s Disease Rating Scale [UPDRS, part III (Fahn and Elton, 1987)], in a practically defined off state (Defer et al., 1999), in fasting condition, before the first intake of dopaminergic drugs in the morning, and 60 min after the oral administration of 250 mg of L-dopa methyl-ester plus 25 mg of carbidopa (UPDRS-III off medication: 25.00 ± 9.51; UPDRS-III on medication: 13.92 ± 6.82). At same time, EEG data were recorded: each session was carried out in eye-closed resting condition.

### EEG recordings

In a sound-proof, temperature controlled room, resting condition eyes-closed EEG data were recorded (*Micromed Brain Quick System Plus Evolution*, Mogliano Veneto, Italy). The EEG recordings were performed (0.3–70 Hz bandpass, notch filtered, linked ear reference) from 19 electrodes positioned according to the International 10–20 System (i.e., Fp1, Fp2, F7, F3, Fz, F4, F8, T3, C3, Cz, C4, T4, T5, P3, Pz, P4, T6, O1; O2). Both the electromyogram (EMG) and electrooculogram (EOG) were recorded, in order to identify any epoch contaminated by artifacts and to, thus, reject it from the analysis. The EMG was recorded from electrodes placed over the submental muscles, with a time constant of 0.03 s. The EOG was recorded by means of bipolar electrodes located about 2 cm above and below the right eye pupil: vertical eye movements were recorded with a time constant of 1 s. Electrode impedance was kept below 5 KΩ. All data were online digitized (256 Hz sampling rate) and stored on a personal computer. Both artefact detection and quantitative analyses were then carried out offline.

During the recording sessions, the participants’ state of vigilance was controlled by visual inspection of EEG traces and subjects’ drowsiness (i.e., slowing of EEG, slow eye movements, appearance of sleep spindles and/or K complexes, etc.). The participants were alerted any time behavioral and/or EEG signs of drowsiness appeared and corresponding EEG traces were not included in the analyses. The EEG epochs with ocular, muscular and other types of artifacts (including those associated with experimenters’ verbal warnings, behavioral and/or EEG signs of drowsiness) were offline visually identified by an expert electroencephalographer, blind to the effective experimental condition, and thus excluded from the analyses. In no case the amount of artifact-free EEG epochs resulted lower than 5 min.

All the signals were analyzed offline using Matlab 2011b (Mathworks, Inc. Natick, MA, USA). During the offline analysis, each EEG recording was divided into 2 s artifact-free epochs that were then submitted to fast Fourier transform (FFT)-based spectral power analysis (Welch technique, Hanning windowing function, no phase shift): power values were calculated at each scalp derivation across a 1–24 Hz frequency range for the following frequency EEG bands: Delta (2–4 Hz), Theta (4–8 Hz), Alpha (8–12 Hz) and Beta (12–24 Hz). Absolute power for each EEG band was then log-transformed before statistical analyses.

### Statistical analyses

Unified Parkinson’s Disease Rating Scale global score and subscale scores [Speech; Facial Expression; Rest tremor of head; Rest tremor of upper extremities (right/left); Rest tremor of lower extremities (right/left); Postural Tremor of hands (right/left); Neck rigidity; Rigidity of upper extremities (right/left); Rigidity of lower extremities (right/left); Finger tapping (right/left); Hand movements (right/left); Pronation-supination movements of hands (right/left); Leg Agility (right/left); Arising from Chair; Posture; Gait; Postural Stability; Body Bradykinesia] were submitted to a Students’ *t* test to compare pre- vs. post- L-dopa administration. Level of significance was set at *p* < 0.05.

A similar model of analysis was applied to EEG data. For each scalp derivation, pre- vs. post- L-dopa administration EEG power values of different frequency bands (Delta, Theta, Alpha, Beta) were compared. To correct for multiple comparisons, the Bonferroni correction was applied: considering the mean correlation between the variables (*r* = 0.57), the alpha level was then adjusted to *p* ≤ 0.006 (corresponding to a *t* ≥ 3.01).

Finally, a correlation analysis was carried out between EEG spectral content and UPDRS scores. More specifically, correlation indices have been computed between the change of each EEG band spectral power (Delta, Theta, Alpha, Beta) and changes of UPDRS scores (Global score, Rest tremor of upper extremities score, Rigidity of upper extremities score, and Body Bradykinesia score), after L-dopa administration (Post-Pre). Electroencephalogram differential values have been calculated independently for each scalp derivation.

## Results

Students’ *t* test on UPDRS scores showed statistically significant improvement of clinical features as a function of L-dopa administration. More specifically, a general improvement of almost all UPDRS part III items was observed (2.14 < *t*_24_ < 13.27; 0.04 < *p* < 0.00000001), except for postural stability and resting tremor.

**Figure 1:**
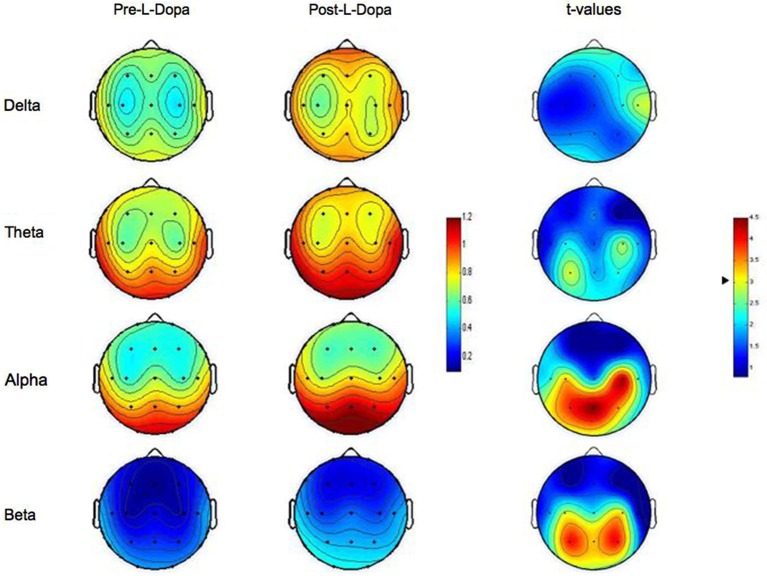
**Topographic statistical distribution of the change in resting wake EEG power, assessed by comparing (*t*-tests) Pre- and Post- L-dopa administration separately for different frequency bands (Delta, Theta, Alpha, Beta)**. Values are expressed in terms of both spectral power (left color-bar) and *t*-values (right color-bar). With respect to *t*-values, the higher the values, the stronger the prevalence of the Post- over the Pre-L-dopa condition. The two tailed level of significance (*p* = 0.006 after the Bonferroni correction, corresponding to a *t* = 3.01) is indicated by the arrow in correspondence of the *t*-values color bar. Average values are normalized by total power, color-coded, plotted at the corresponding position on the planar projection of the scalp surface and interpolated between electrodes. The maps are based on the 19 unipolar EEG derivations of the international 10–20 system with averaged mastoid reference.

The analysis carried out on EEG power spectral content showed differential effects for each EEG band with marked topographical differences. As depicted in Figure 1, no statistically significant differences arose on Delta and Theta bands as a function of L-dopa administration.

Conversely, after the intake of L-dopa, the Alpha rhythm showed a sharp increase on centro-temporo-parietal scalp derivations. More specifically, a significant increase in Alpha band was observed on C3 and C4 (*p* = 0.003 and *p* = 0.0002, respectively), T5 (*p* = 0.005), and P3, P4 and Pz (*p* = 0.0007, *p* = 0.002 and *p* = 0.0002, respectively). No other recording sites reached the statistical significance.

On the same way, also the analysis on Beta band indicated a significant increase of EEG spectral power after L-dopa ingestion. Again, such an effect was localized and limited to centro-parietal areas and particularly to C4 (*p* = 0.006), and P3, P4 and Pz (*p* = 0.0006, *p* = 0.0006 and *p* = 0.003, respectively). No other recording sites reached the statistical significance.

Correlation analysis indicated some significant effects, once more circumscribed to centro-parietal areas and to faster rhythms. Alpha band increase on C4 after L-dopa intake negatively correlated with Rest tremor of arms (*r* = −0.42, *p* = 0.039). No other significant correlations were observed. In the same vein, the increase of Beta spectral power in C3 and C4 negatively correlated with Rigidity of arms (*r* = −0.46, *p* = 0.025; and *r* = −0.43, *p* = 0.033, respectively) and Bradykinesia (*r* = −0.45, *p* = 0.027; and *r* = −0.52, *p* = 0.0097, respectively). Also, Beta increase in P3 negatively correlated with Rigidity of arms (*r* = −0.48, *p* = 0.017), while Beta increase in P4 with Rigidity of arms (*r* = −0.51, *p* = 0.011) and Bradykinesia (*r* = −0.43, *p* = 0.038).

## Discussion

In its classical model, PD is considered a predominantly subcortical disease, involving several structures in the brainstem and the diencephalon. However, different EEG studies found that cortical oscillatory resting activity is altered even in the early stages of the disease, when cortical layers are supposed to be anatomically spared. This supports the hypothesis that basal ganglia-thalamo-cortical circuitry is extremely involved in the generation of physiologic cortical EEG rhythms. If we assume that EEG modifications (i.e., widespread slowing) in PD are due to a disruption in thalamo-cortical circuits, we might expect that dopamine replacement treatment, by re-establishing a more physiological thalamo-cortical coupling, will reverse the EEG slowing. Therefore, we conducted a study aimed to investigate the effect of DRT on the oscillatory brain activity of cognitively preserved Parkinsonian patients. In particular, we analyzed eye-closed resting state activity and quantitative EEG modifications before and after an acute L-dopa administration, and found that L-dopa determines a significant modification of EEG power spectrum, leading to a significant increase of alpha and beta power. Alpha and beta frequencies constitute the leading characteristic of normal EEG activity at rest, frequently referred to as the “idling rhythms” of the adult brain (Niedermeyer and Lopes da Silva, 2004). Modern theories support the hypothesis that spontaneous alpha activity is more than a passive rhythm, but, possibly, a deterministic chaotic signal with several functional correlates (Basar et al., 1997; Schürmann and Basar, 2001), working as a basic form of information transmission in the brain (for a review see Klimesch, 1999). Given these premises, the disruption of spontaneous alpha activity within a determined cortical area might be interpreted as an EEG marker of altered cortical functioning and processing of information; conversely, an alpha rhythm increase on resting EEG may be considered as the signature of a restored normal cortical oscillatory activity, affecting the production and conduction of signaling in the brain and facilitating integrative functions (Basar, 1990; Basar et al., 1997).

Similar assertions might be claimed for beta frequency band. Beta rhythm is widely recognized to be linked with motor behavior and response inhibition, top-down signaling associated with selective attention (Gross et al., 2005), working memory (Tallon-Baudry et al., 2001), guided search (Buschman and Miller, 2007), object recognition (Sehatpour et al., 2008), perception (Donner et al., 2007) or sensorimotor integration (Brown and Marsden, 2001; Brovelli et al., 2004; Witham and Baker, 2007; Lepage et al., 2008). Considering its wide involvement, beta power increase may indicate an improvement in cerebral integrative and motor functions, further supporting the motor “idling” hypothesis (Pfurtscheller et al., 1996).

Based on these results, and assuming that a significant modification in EEG power spectrum after L-dopa intake may be, at least in part, related to dopaminergic mechanisms in its generation, it may be hypothesized that dopaminergic defective networks are implicated in resting-state cortical oscillatory abnormalities in PD patient even before dementia occurs, and may be reversed by L-dopa administration.

Interestingly, the effect on EEG power spectrum of L-dopa administration was circumscribed to well-defined cortical areas, mainly centro-parietal regions. The power increase of alpha and beta rhythms spared anterior (i.e., frontal) derivations, suggesting that L-dopa influence on cortical oscillatory activity, in the early stages of PD, may be conveyed mainly through dopamine depleted nigro-striatal-thalamo-cortical networks, rather than through direct dopaminergic mesocortical pathways projecting to frontal cortex.

These findings display clinical implications: our correlation study investigating the relationship between EEG power spectrum modifications and patients’ motor performances found that an improvement in specific motor subtests of the UPDRS significantly correlated with an increase in beta power spectrum on centro-parietal derivations. The more motor performances improved, the more beta power increased on the above-mentioned regions. A minor correlation effect was also found for alpha power and resting tremor. In other words, beta and alpha power increase on centro-parietal regions was not a simple epiphenomenon, but correlated with motor improvement, further sustaining the hypothesis that EEG slowing in non-demented PD patients is related to dopaminergic defective networks strictly involved in motor control.

The currently accepted view on the pathophysiology of PD primarily involves a dysfunction of the basal ganglia circuits, which determines a reduced excitatory thalamic outflow, and, eventually, a hypoactivity of several cortical areas, i.e., the primary motor cortex and other non-primary motor areas (Dick et al., 1989; Catalan et al., 1999; Sabatini et al., 2000). Consequently, our finding that an increase of beta and alpha power on central derivations after a L-dopa load correlates with an improvement in motor performances, may reflect a L-dopa-related restoration of an appropriate motor cortex oscillatory working, sustained by a re-established thalamo-cortical coupling. The effects on cortical activation might be important in terms of motor response and motor programming. Moreover, the effect revealed on parietal circuitry suggests that the improvement in motor performance may be also related to the “sensory” aspect of motor control. The parietal cortex plays a very important role in sensorimotor integration (Chersi et al., 2011). This process may be altered either by abnormalities in the peripheral afferent input or by an aberrant central response to sensory input. A deficit in sensorimotor integration is well established to occur in PD (Flowers, 1976; Schneider et al., 1987; Klockgether and Dichgans, 1994; Rickards and Cody, 1997; Fellows et al., 1998; Gerschlager et al., 1999) and neurophysiological studies suggest that the alteration depends on aberrant processing of sensory signals at a cortical level (Rossini et al., 1989, 1998; Degardin et al., 2009). Although highly speculative, we suppose that a dopaminergic mechanism may be implicated in the deterioration of sensorimotor integration that occurs in PD, and that L-dopa, by restoring, at least partially, normal patterns of oscillatory activity within the basal ganglia, may alter the circuitry of the parietal cortex, allowing an implemented integration of sensory inputs at a cortical level and, consequently, a correct processing of motor programs. Unfortunately, the present study did not directly assess proprioceptive sensitivity.

At first glance, our study might seem in disagreement with other studies claiming for an inverse correlation between synchronization in beta frequencies and motor aspect of PD. Different research groups found an association between elevated cortical and subthalamic beta-band synchrony and bradykinesia, as well as a correlation between dopaminergic medication or subthalamic stimulation, decreased beta-band synchrony and improvement in motor performances (Brown and Marsden, 1999; Doyle et al., 2005). Nevertheless, our study focuses on resting EEG activity and explores oscillatory brain functioning in a state that is far different from event-related modification of EEG power spectrum, but it is surely a stable and interpretation-free condition reflecting changes in a “default” network which is known to be affected in many widespread diseases of the brain, e.g., PD (Bosboom et al., 2006), multiple sclerosis (Cover et al., 2006) and Alzheimer’s disease (Stam et al., 2006).

Future studies would investigate functional brain connectivity as a consequence of DRT, assessed during different motor actions, in order to evaluate the effective changes in brain functioning after a L-dopa dose.

## Author contributions

Jean-Marc Melgari conceived the study, carried out the data collection, contributed to data analysis and statistical analysis, played a major role in the general idea of the paper and wrote the paper. Giuseppe Curcio analyzed the data, performed statistical analysis and helped to write the paper. Francesca Mastrolilli carried out the data collection, contributed to data analysis and statistical analysis. Gaetano Salomone carried out the data collection, contributed to data analysis and with the general idea of the paper, and helped to write the paper. Laura Trotta carried out the data collection, contributed to data analysis and helped to write the paper. Mario Tombini contributed to interpret the results and critically revised the paper. Lazzaro di Biase carried out the data collection and contributed to data analysis. Federica Scrascia carried out the data collection and contributed to data analysis. Rita Fini carried out EEG data collection. Emma Fabrizio carried out EEG data collection. Paolo Maria Rossini helped with the general idea of the paper, contributed to interpret the results and critically revised the paper. Fabrizio Vernieri helped with the general idea of the paper, contributed to statistical analysis, to interpret the results and critically revised the paper. The work presented here was carried out in collaboration between all authors. All authors read and approved the final manuscript.

## Conflict of interest statement

The authors declare that the research was conducted in the absence of any commercial or financial relationships that could be construed as a potential conflict of interest.
